# Industrial Shared Wireless Communication Systems—Use Case of Autonomous Guided Vehicles with Collaborative Robot

**DOI:** 10.3390/s23010158

**Published:** 2022-12-23

**Authors:** Jacek Stój, Anne-Lena Kampen, Rafał Cupek, Ireneusz Smołka, Marek Drewniak

**Affiliations:** 1 Department of Distributed Systems and Informatic Devices, Faculty of Automatic Control, Electronics and Computer Science, Silesian University of Technology, 44-100 Gliwice, Poland; 2 Department of Computer Science, Electrical Engineering and Mathematical Sciences, Faculty of Engineering and Science, Western Norway University of Applied Sciences, 5020 Bergen, Norway; 3 AIUT Sp. z o.o. (Ltd.), 44-109 Gliwice, Poland

**Keywords:** AGV, CoBotAGV, M2M, converged networks, real-time system, real-time ethernet, Profinet, OPC UA, MES, wireless communication, Wi-Fi

## Abstract

Dedicated fieldbuses were developed to provide temporal determinisms for industrial distributed real-time systems. In the early stages, communication systems were dedicated to a single protocol and generally supported a single service. Industrial Ethernet, which is used today, supports many concurrent services, but usually only one real-time protocol at a time. However, shop-floor communication must support a range of different traffic from messages with strict real-time requirements such as time-driven messages with process data and event-driven security messages to diagnostic messages that have more relaxed temporal requirements. Thus, it is necessary to combine different real-time protocols into one communication network. This raises many challenges, especially when the goal is to use wireless communication. There is no research work on that area and this paper attempts to fill in that gap. It is a result of some experiments that were conducted while connecting a Collaborative Robot CoBotAGV with a production station for which two real-time protocols, Profinet and OPC UA, had to be combined into one wireless network interface. The first protocol was for the exchange of processing data, while the latter integrated the vehicle with Manufacturing Execution System (MES) and Transport Management System (TMS). The paper presents the real-time capabilities of such a combination—an achievable communication cycle and jitter.

## 1. Introduction

Autonomous Guided Vehicles (AGV) are becoming a common solution in manufacturing systems [1,2]. The increased interest in AGV is associated with the agile production paradigm, which is based on a dynamic chain of manufacturing services. The items that are transported by AGV must be loaded and unloaded. This takes time and labor. The best way to automate this task is to use a robot, which can be placed right on an AGV. Importantly, it has to operate in collaboration with both the production stations and personnel. Such an AGV with an integrated robot is typically called a CoBotAGV. For simplicity, they will be referred to as AGV. Cooperative robots function as production-station extensions that require real-time communication with other components of the control system, while other scenarios require soft real-time communication. For instance, very close cooperation between AGV and the Manufacturing Execution System MES is required when there are deadlocks between several moving AGVs [3,4]. AGVs not only operate as a transport system but also as a mobile storage space that must take into account the availability of production stations, which in classic production systems is solved by using hardware, e.g., the length of a conveyer belt or the number of hangers.

Modern industrial IT systems are supported by technologies such as machine learning (ML), artificial intelligence (AI), and Big Data, all of which require that big sets of data be collected in order to generate intelligent decisions [5,6]. AGVs are valuable data sources for all of these systems [7]. This also results in the transmission of a large volume of data. This has caused a shift from point-to-point communication with AGV, which is based on a well-defined information format [8], to a mesh communication topology that has different systems that have dynamically established communication services. Considering the communication of AGV with an MES or a TMS (both of which are parts of business intelligence BI) or other data analytics systems such as the systems that are used for predictive maintenance or energy management, data can usually be delivered in a batch manner without significantly affecting the operation of these systems. This enables large-volume data to be buffered for transmission once a high bandwidth and reliable communication link have been established. Thus, real-time, soft-real-time, and batch communication requirements should be considered when planning communications with AGV. The need for wireless communication protocols to integrate and cooperate is an enabling factor for smart transportation systems including both drones (Unmanned Aerial Vehicles) and wheeled vehicles including the AGVs that are discussed in this paper [9].

The communication scenarios described above result in new requirements for communication with AGV that considers (i) the different characteristics of the communication traffic, including the different requirements for throughput, reliability delays, and transmission jitter. The communication requirements that apply when an AGV is docking to a production station or when a cooperative robot is performing production tasks differ from the requirements that apply when an AGV is communicating with an MES. In the first case, AGV should be treated as an element of the control system (often working on the hard real-time principle). In the second case, the soft real-time communication paradigm is usually sufficient and (ii) communication with different systems requires different protocols. In control systems, fieldbus communication protocols or Industrial Ethernet protocols can be used, while in MES, a service-oriented communication is typically used, (iii) communication with AGV is a wireless communication that in most cases has variable parameters, which depend on both the availability of wireless networks, i.e., the location of the AGV and on the load on these networks, which varies over time and is highly unpredictable. Even when a separate wireless communication infrastructure is dedicated to AGV, it is challenging to predict the number of AGVs and the required communication services that will be required at a given access point at a given moment. The reason for this is the flexibility of logistics tasks, i.e., it is challenging to predict the location of moving AGV [10], do the path planning [11] and, in addition to communicating with an MES for the task allocation. among other things [12]. Importantly, CoBotAGV must also wirelessly integrate with the production stations to enable the actual robot collaboration.

In research for this paper, we experimentally investigated the wireless communication between AGV and production stations as well as wireless communication between AGV and an MES. Profinet was used to communicate with the production station. OPC UA was used to communicate with the MES. Our aim was to meticulously investigate to what degree Profinet real-time communication was affected by the OPC UA-traffic. The findings are not only valuable for Profinet-OPC systems, but also for similar communication systems.

Profinet is one of the most popular protocols that is used in industrial applications (See: https://www.hms-networks.com/news-and-insights/news-from-hms/2022/05/02/industrial-networks-keep-growing-despite-challenging-times (accessed on 28 October 2022)) [13] and can be used to communicate directly with robots such as Universal Robots (See: https://www.universal-robots.com/ (accessed on 13 September 2022)). The OPC UA protocol is probably the most widely used protocol in industrial scenarios for integrating the devices on the factory floor with manufacturing systems.

### 1.1. The Concept of a CoBotAGV Wireless System

**Figure 1:**
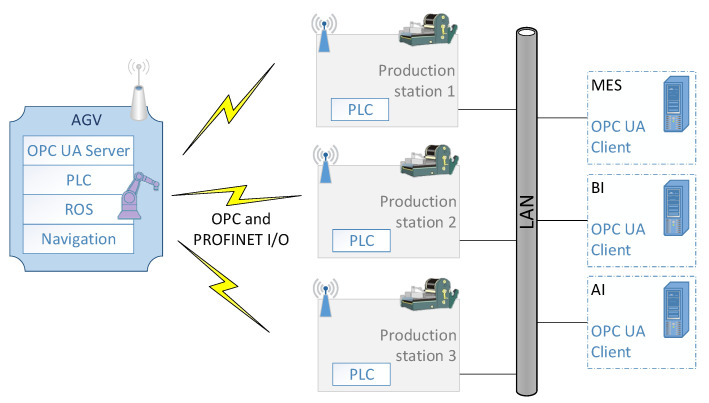
Industrial infrastructure with an AGV system, OPC UA and Profinet.

The concept of the communication system is presented in Figure 1. On the left side of the figure, there is a single AGV that has some components installed on it: (a) a programmable logic controller PLC with some I/O modules to control the AGV; (b) an ROS to control the collaborative robot; (c) a navigation system and (d) an OPC UA Server for collecting information about the state of the AGV and the robot. In the middle of the figure, there are three production stations to which the AGV docks.

The AGV needs to exchange some data with the station to enable the robot to operate. The production stations are connected to a LAN through which the higher-level components of the system are available (here: a Manufacturing Execution System (MES)). There are also some OPC UA clients that need the data that is collected on the AGV.

### 1.2. Main Contribution

The contribution of the paper is three-fold: (i) to discuss the communication services for an AGV within the scope of communicating with control systems and an MES; (ii) to analyze and investigate the impact of OPC UA communication on Profinet communication when using the same medium and (iii) to identify the characteristics of wireless communication that are important to address in order to meet the requirements of different applications.

The results of the experimental research showed the impact of a communication process that is realized using the OPC UA protocol on the data exchange process using the Profinet protocol in a wireless network. As a benchmark, the optimum data exchange cycle was determined for Profinet communication when no other protocols that were generating traffic were present. Then, the communication jitter was measured when OPC UA was introduced into the network while sending different amounts of data with a different number of clients.

### 1.3. Organization of the Paper

The rest of the paper is organized as follows: Section 2 presents the Profinet protocol and emphasizes the relationship between the parameters for cyclical exchanges and the occupancy of a communication medium. The Profinet components such as the specific switches for real-time communication are also identified. Section 3 presents the principles of information exchange in OPC UA with an emphasis on the relationship between the session and subscription parameters and the traffic in the communication channel. In the presented research, OPC UA is used to communicate with both the production execution systems and the analytical systems for batch data transmission. Section 4 discusses the properties of the wireless communication protocol and analyzes their impact on both the average throughput and the maximum variation in the delay (jitter). The parameters of wireless communication are the key when analyzing the network transmission parameters for both the Profinet I/O and OPC UA communication. Section 5 presents the results of the experimental research and compares them with the theoretical predictions. The conclusions are presented in Section 6.

## 2. Related Works

Whenever the future of industrial systems is discussed, the OPC UA protocol is mentioned [14]. There is a wide range of application scenarios for the OPC UA protocol in industrial systems. One example is described in [15], where it is used to integrate a process network consisting of a programmable logic controller, with the Industrial Internet of Things IIoT. Another example of this kind of OPC UA application is presented in [16]. Generally, integrating different communication systems in industrial systems is recognized as the main goal in future process automation [17].

The convergence of Profinet and the OPC UA protocol was discussed in [17]. However, here, while an IT network was integrated with an OT network these two protocols were assigned to separate VLANs. It was also stressed that the performance and characteristics of the real-time system must be preserved. The idea of IT and OT network convergence is also presented in [18].

The coexistence of OPC UA with other protocols is presented in [19]. Here, MQTT is discussed and its feasibility is shown. However, the temporal aspects are not addressed. Some performance analysis of OPC UA is presented in [20], but once again, in reference to MQTT.

The performance of OPC UA, Profinet, and some other protocols such as EtherCAT and Ethernet Powerlink are compared in [21]. However, the protocols are only investigated when operating separately, i.e., one protocol in a network at a time. We have not found any publications in which both protocols are simultaneously included in one common wireless network.

The OPC UA and Profinet protocols are sometimes grouped into one network using the same physical communication interface (Ethernet) although not as a separate solution but as a coupled solution. It is possible to use OPC UA to meet the requirements of autoconfiguring Profinet network devices as discussed in [22]. However, the issue of the influence of OPC UA protocol on the temporal parameters of a Profinet network is not addressed.

## 3. Profinet Protocol

Profinet is one of the most common protocols that are used in Ethernet-based networks (According to: https://www.automation-fair.com/2021/04/12/industrial-ethernet-continues-to-gain-market-share-ethernet-ip-and-profinet-strive-for-first-place/ (accessed on 28 August 2022)) [23]. Communication in Profinet is performed according to the consumer-provider communication principle. It is designed for use in industrial systems where process data are exchanged between communication network nodes such as programmable logic controllers or input/output stations. The data exchange is typically realized between one IO-Controller and one or more IO-Devices. The cycle of the real-time data exchange can be below 1 ms [24]. The IO-Controller is responsible for starting up the network and coordinating the data exchange. The user data, i.e., the process data is transferred in a cyclic manner according to the update time parameter, which is defined independently for each IO-Device. The parameter is used to maintain the required industrial process operation as it individually decides the frequency of data exchange with each IO-Device. It should also fit the controller automata cycle of the Profinet IO-Controller, which is usually implemented as a PLC controller, e.g., Programmable Logic Controller—PLC.

The PLC controller executes a user program that processes the input and output data exchange in the communication network. As a rule, this is completed in a cyclic manner. One program execution is called an automata cycle or simply a cycle. There is no reason to send the process data (input/output data) more often than it is needed from the program execution point of view, e.g., when the automata cycle is 20 ms long, sending the process data with 1 ms cycle is too frequent and leads to a throughput waste of the communication network.

The cyclic data transmission is performed in real-time by the Profinet IO protocol, which is specified at the application layer of the ISO/OSI reference model. In the lower layers, i.e., the data link layer and physical layer, the Ethernet protocol is used.

Process data is not the only type of data that can be transmitted in Profinet networks. To support network startup and operation, configuration data, diagnostic data, etc., are also transmitted using the TCP/IP protocol stack. However, this communication is not real-time. In addition, other protocols (not associated with Profinet and process data transmission) can use the same physical infrastructure. Thus, the infrastructure is not dedicated exclusively to Profinet communication (as it would be in some other networks such as EtherCAT). Therefore, it is possible to provide many different services in addition to the Profinet communication, e.g., access to the web servers of the network devices using the TCP/IP protocol stack. In our case, the OPC UA protocol exchanged data with the MES across the Profinet network.

### 3.1. Profinet Real-Time Operation

Data transfer must be performed in a timely manner in industrial applications. Datagram delivery-delay must be temporarily determined and the jitter must be kept at an appropriate level. Three real-time classes are defined in a Profinet network, and two of those provide real-time communication: RTC1 and RTC3. There is also an RTC2 class, however, it is not used in the newer applications and is considered to be obsolete.

In the RTC1 class (also referred to as a Real-Time class or simply an RT class), the typical update times are about dozens of milliseconds and the jitter is between 15% to, at most, 25% of the cycle time. In the RTC3 communication class, which is referred to as Isochronous Real-Time communication (IRT), both the typical update time and jitter are quite low, 1 ms and 1µs, respectively [25]. Such a low jitter is the result of the synchronous transmission of datagrams between the communication system nodes, which is performed in constant cycles according to strict rules and with the datagrams prioritized [26].

In order to maintain Profinet traffic in an IRT class with these temporal parameters, it is necessary to use dedicated hardware that is equipped with ASIC (application-specific integrated circuits). In this paper, wireless communication is considered, which cannot be accomplished in an RTC3 real-time class (IRT).

Communication in RTC1 is possible although not recommended by PI (Profibus & Profinet International: https://www.profibus.com/pi-organization (accessed on 20 December 2022)) for wireless communication in industrial scenarios. However, wireless communication is sometimes indispensable, for instance, for communication between an AGV and its environment. Therefore, Profinet communication in the RTC1 using wireless interfaces was investigated in the research for this paper. In addition, since AGV not only communicates via Profinet but also via OPC UA, in our experimental scenario, the effect of the OPC UA communication on the Profinet temporal characteristics had to be determined.

### 3.2. Profinet Network Startup and Communication

During the startup of a Profinet network, the IO-Controller sends identification requests to the IO-Devices using their network names and a Dynamic Configuration Protocol (DCP). Together with the IO-Device response, the IO-Controller gets the MAC address of the responding device. Once it has the MAC address, the IO-Controller configures the IO-Devices—sets their IP addresses and other configuration parameters using the Context Management protocol (CM) after which the real-time communication begins.

The IO data is exchanged between the IO-Controller and IO-Devices in a cyclic manner according to the update time (defined for each device separately). Each transmitted datagram is numbered with a cycle counter. By default, when no new datagrams arrive within a given period, which is defined as the triple update time, a timeout occurs, and the communication is broken. The number of cycles without any IO data (default: 3) can usually be adjusted to the requirements of the application. Communication can be established again by performing the startup procedure (using the DCP and CM protocols). Due to the unreliability of wireless links, the number of cycles that are allowed to pass without any IO data must be carefully determined. A value that is too high increases the delay in detecting a fault. A value that is too low can cause breaks in communication and require that a new connection be established (using the DCP and CM protocols as mentioned above).

### 3.3. Wireless Profinet Network

Using Profinet, the IP protocol is only required to configure and diagnose the IO-Devices. The network startup is based on the device names [27], while the real-time data exchange is based on the MAC addressing. The typical IEEE 802.11 (Wi-Fi) access point (AP) cannot be used to bridge the wired-wireless part of the network because the MAC addresses in the Ethernet datagrams are changed while traversing the AP. Instead, ‘transparent’ wireless bridges that do not alter the Profinet datagrams are required. Such a connection can be made with a Wireless Distribution System (WDS) [28], which is the method of wireless communication that was used during this experimental research.

## 4. Object Oriented Communication Middleware for Agile Manufacturing

One of the problems that are common in agile manufacturing is the need to dynamically create communication channels between various resources such as machines, production stations, control equipment, etc., as well as to dynamically create communication paths between the various systems that provide or coordinate the IT services for production support such as scheduling and dispatching, internal logistics management, production tracking, performance analysis, or production maintenance [29]. On the one hand, success is determined by the short time required to create a new communication channel between machines or systems and on the other hand, by minimizing the risk of any errors that are made when establishing such a connection. One of the ways of unifying machine-machine and machine-system communication is to replace the direct communication interfaces with a universal interface that is based on the concept of communication middleware [30].

Communication middleware is an intermediary layer whose task is, on the one hand, to standardize the interface in terms of access to the data and services and on the other hand, to develop schemas that are based on object-oriented methods that provide not only data but also additional information that describes the data that is available as metadata. Metadata can describe either the technological meaning of a process variable, the properties of the devices, or the technological processes as well as the properties of the communication interfaces that enable the optimal use of the combination of the communication channels that are available in a given cooperation context. The advantages of communication middleware are its flexibility and universality, which are obtained at the cost of the transmission time parameters [31]. For this reason, such a solution is mainly used for the soft real-time or non-real-time communication that is typical for SCADA, MES, or BI applications, while for hard real-time communication, dedicated protocols such as Profinet I/O are used. In many cases, both types of protocols must share a common communication medium.

OPC UA is an example of object-oriented and service-based communication middleware that is standardized by the OPC Foundation (See: https://opcfoundation.org (accessed on 20 December 2022)). It fulfills the different communication needs that were supported by previously dedicated communication protocols. It enables both the presentation of meta-models and physical information exchange. OPC UA is defined by abstract services that are implemented by several OPC UA software stacks that can be used for the software applications created in programming languages such as C++, C#, Java, or Python. OPC UA communication can be based on the opc.tcp protocol (an extension of TCP/IP with additional security features) or based on the protocols that are used for web services. In this research, both opc.ip communication and the C# OPC UA stack, which are supported by Unified Automation, were used.

OPC UA communication is based on the client-server model. The server provides several services (the actual set depends on a server’s profile) that are available to clients. All of the services are defined on an abstract level, which means that they are independent of the communication protocol or programming stack and enable seamless work in a heterogeneous environment. The services are grouped into sets according to the functionality that they offer: (i) a Discovery Service Set, a SecureChannel Service Set, and a Session Service Set, which support finding the required OPC UA server and establishing safe communication between an OPC UA client and the server; (ii) a View Service Set and a Query Service Set, which are used for discovering the information that is available on the server and its model as expressed by the nodes and references that are used for the object-oriented modeling; (iii) a Node Management Service Set, which is used to manage the nodes and references that are exposed by the server; (iv) an Attribute Service Set that provides direct access to the attributes of the nodes; (v) a Method Service Set, which enables the routines that are offered by the objects that are available on the server to be used and (vi) a Subscription Service Set and a MonitoredItem Service Set, which are used to establish and manage the communication channels that are created between the OPC UA Clients and the OPC UA Server. The last (vi) group of services not only enables the available variables to be selected but also enables parameterization of the communication channels, which is directly related to the research scope of this paper.

**Figure 2:**
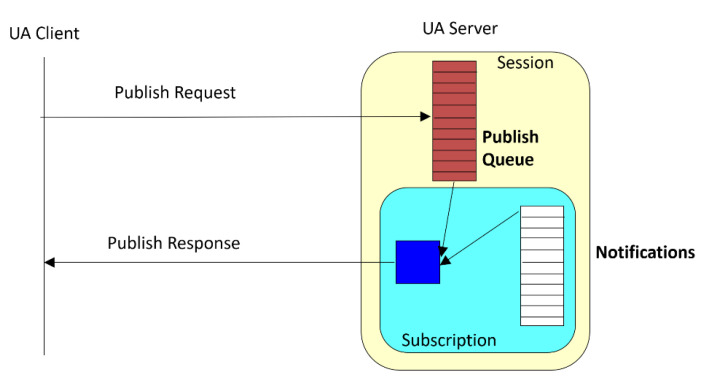
Subscription-based communication in OPC UA.

The subscription-based communication services enable the transition from the communication mode query response (request confirmation), which is a typical client-server communication, to the producer-consumer communication model in which the server acts as the data producer in accordance with the parameters that are required in the subscription by a given client [32]. This model differs from the consumer-provider model that is used, e.g., in the Profinet I/O in that the client must periodically send a Publish Request to which the server responds with a Publish Response as is presented in Figure 2. Such a cyclic request is necessary because the underlying communication protocols that are used by OPC UA do not allow callback communication. The advantage of a subscription is a significant reduction in the volume of information that is exchanged via the network; the disadvantage is that the stateful communication mode is used in which all of the subscription parameters have to be stored on the server [33].

After the server is found and the client is authenticated, the next session is created according to services that are supported by the first (i) group. From this moment, each session forms a logical connection between the OPC UA client application and the OPC UA server application. Next, one or more session(s) can be created and activated by the client. There is a set of parameters that have to be individually determined for each subscription [34]. In this paper, we focus on only three parameters: requestedPublishingInterval, requestedLifetimeCount, and requestedMaxKeepAliveCount, all of which have a direct impact on the communication traffic that is visible in the physical layer of the network.

The requestedPublishingInterval parameter defines the cyclic rate that the Subscription is being requested to use for the return Notifications to the Client. This interval is expressed in milliseconds. It is represented by the publishing timer in the Subscription state table and can be negotiated between the client and server. This parameter directly affects how often the client will send a Publish Request to the server. When new information that is required by a subscription is available on the server, a Publish Response with the new data will be performed.

The requestedMaxKeepAliveCount parameter determines the maximum keep-alive counter value. When the publishing timer expires this number of times without requiring any NotificationMessage to be sent, the Subscription sends a keep-alive Message to the Client. This parameter also determines the maximum period that can elapse before the server must send a Publish Response to the Client even if it has no new data. Thus, these two parameters define the minimum and maximum time of the information cycle for a given subscription.

The requestedLifetimeCount parameter determines the maximum value for the lifetime counter. The Subscription is deleted by the Server if the number of consecutive undelivered requests exceeds the maximum counter. The value of this parameter, on the one hand, is related to the resistance of a given subscription to communication delays (a higher value enables it to work with a less stable network in terms of jitter). However, on the other hand, a value that is too high increases the time after which any communication breaks are detected.

**Figure 3:**
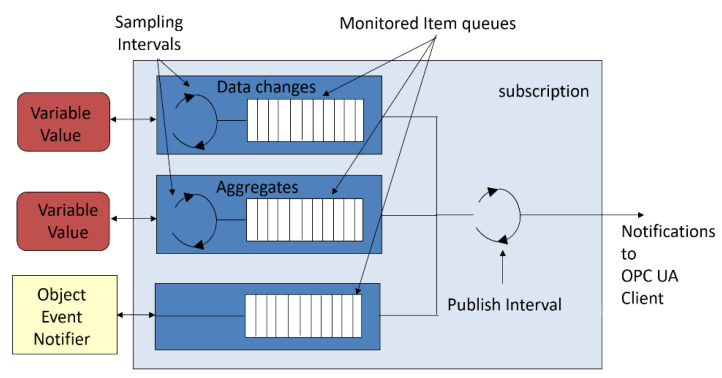
Data buffering in subscription-based communication in OPC UA.

The information content that is sent on a given subscription depends on the requirements that were declared by the Client using the services, which are grouped by the MonitoredItem Service Set. Each variable that is transmitted in a subscription can be sampled at a different frequency. Individual samples are buffered and then sent together with the other variables that were declared for a given subscription as is shown in Figure 3. Cyclic sampling can be used for both the raw process data values as well as for the aggregates, e.g., the average or maximum value for a given period. In the case of events, the queue is also used, but due to the unpredictable nature of an event, its impact on communication traffic cannot be precisely calculated. In our experimental tests, only the first type of transmission—the cyclically read values of the variables with buffering by consistently sized queues were used.

## 5. Wireless Communication

Wireless communication is one of the most popular methods for exchanging data between devices that are commonly used [35]. In our daily life, it is used to access the Internet, stream multimedia and use GSM. Additionally, wireless devices have become more important in industrial environments, and are one of the fundamental components of Industry 4.0 [36]. The multitude of available solutions enables devices to be matched and used in any environment, while the data rate that is provided continuously increases. Our experimental tests assessed the end-to-end delay and jitter for solutions that are based on a Wi-Fi interconnection.

The typical frequencies that are used for wireless industrial networks are 2.4 GHz (included in the reserved radio spectrum for Industrial, Scientific and Medical purposes) or 5 GHz, although there is already a newer 6 GHz radio spectrum [37]. The different frequencies have different characteristics. Higher frequencies provide increased bandwidth, and consequently an increased data rate. However, the transmission range is shorter than for the lower frequencies. All wireless RF transmission is susceptible to signal disruption due to EM interference from the environment. In addition, obstacles or even dust can lead to signal attenuation and reflections, for which the impact is more severe for a higher transmission frequency. The following expression, which is presented in [38], can be used to analytically estimate the signal loss in free space:

(1) $$L_{f}=20\log_{10}\left(d\right)+20\log_{10}\left(f\right)-x$$

*L_f_*—FSPL—Free Space Path Loss
*d*—distance between the antennas in meters
*f*—frequency of the signal in MHz
*x*—propagation constant to “*d*” in meters and “*f*” in megahertz (−27, 55)

Example:

(2) $$L_{2.4\mathrm{GHz}}=20\log_{10}\left(5\right)+20\log_{10}\left(2400\right)-27.55\approx 14+67.6-27.55\approx 54.05\left[\mathrm{dB}\right]$$

(3) $$L_{5\mathrm{GHz}}=20\log_{10}\left(5\right)+20\log_{10}\left(5000\right)-27.55\approx 14+40-27.55\approx 60.43\left[\mathrm{dB}\right]$$

The popular Multi-Wall model can be used for propagation with obstacles, Table 1 lists the factors that describe the materials as well as the calculations that were used.

(4) $$L=L_{0}+10\gamma \log\left(d\right)+\overset{x}{\underset{i=1}{\sum }}mL_{w}+\overset{x}{\underset{i=1}{\sum }}nL_{c}$$

*L*—attenuation between antennas [dB],
*L*_0_—attenuation referenced to 1 m distance in [dB],
*L_w_*—attenuation for a wall [dB],
*L_c_*—attenuation for a ceiling [dB],
*γ*—power loss index (2 for free space, from 3.5 to 6 for space with obstacles),
*d*—distance between the antennas in meters,
*m*—number of walls,
*n*—number of ceilings.

These equations enable the approximate loss of radio signal to be calculated and the appropriate devices and transmission parameters to be determined.

In addition to attenuation, power loss, obstacles, and other issues that were mentioned above, wireless communication is affected by interference from co-located networks that are communicating at the same frequencies. In our case, it not only applied to other IEEE 802.11 (Wi-Fi) networks, but also to other standards that were using the ISM band such as ZigBee, which is based on IEEE 802.15.4 [39,40] or Bluetooth (IEEE 802.15.1) [41,42].

Although they are not a part of the contribution of this paper, security and safety are very important in industrial networks in order to protect the data as well as the network infrastructure [43]. Successful malicious attacks can lead to the loss of revenue, loss of credibility, loss of production, etc., which can be devastating for a manufacturing company. There are many examples of successful attacks. The most well-known is most likely stuxnet [44,45].

## 6. Experimental Research

Experimental research was conducted to evaluate to what extent a wireless channel can be shared between the OPC UA and Profinet protocols without violating the real-time constraints.

### 6.1. The Testbed

**Figure 4:**
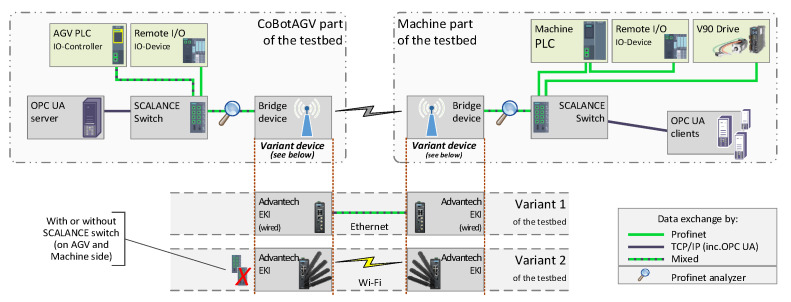
Profinet and OPC UA convergence—the two variants of the experimental research testbed: Variant 1: wired communication; Variant 2: wireless communication.

The testbed was divided into two parts: the AGV part and the Machine part as is presented in Figure 4. On the AGV side, there was an industrial programmable logic controller (PLC) that was responsible for operating the AGV (controlling the servo drives and operating the collaborative robot, i.e., cooperating with the machine, safety functions, etc.). The AGV PLC operated as a Profinet IO-Controller with four IO-Devices: one remote I/O station, which was installed on the AGV side, and three more devices on the Machine side (a servo drive, a remote I/O station and a machine PLC, which was operating as an I/O Device).

On both the AGV and the Machine side, there were industrial SCALANCE X208 Ethernet switches, which provided the connection between all of the Ethernet devices on each side. Additionally, the SCALANCE switches were connected together by Advantech EKI-6333AC-4GP switches that were equipped with a wireless interface. These provided the connection between the AGV and the machine in three ways:

- via a wired Ethernet 1 GB/s connection (the 1 GB/s was only between the EKI switches; all of the Profinet devices used 100 MB/s), which was Variant 1 of the testbed,
- via a wireless IEEE 802.11b (2.4 GHz) connection (variant 2 of the testbed),
- via a wireless IEEE 802.11n (5 GHz) connection (variant 2 of the testbed).

Moreover, in the latter part of the experimental research, the SCALANCE switches were removed, and all of the devices were connected directly to the EKI switches.

To determine the quality of the communication in the Profinet network, an analyzing tool was used—Profinet-Inspector NT by Indu-Sol. It was placed between the SCALANCE and the Advantech EKI switches. The “magnifying glass” image in Figure 4 shows its location.

### 6.2. Profinet Inspector

The Profinet Inspector is a diagnostic tool that is designed to diagnose Profinet networks. It can be placed between any two Ethernet devices. It operates akin to a Terminal Access Point (TAP), which means that it is transparent to the Ethernet communication and does not influence the flow of Ethernet datagrams. It captures the datagrams and analyzes the Profinet network operation. In addition, it detects any Profinet devices and their settings, e.g., the update time. It measures jitter, detects any failures of the Profinet devices, checks for any frame gaps, and captures information about the alarms that are sent between the Profinet devices. Moreover, it can read the statistical data from the switches, e.g., their payload and the number of erroneous datagrams that are received.

### 6.3. Profinet Update Time Considerations

As a benchmark, a measurement was first performed on a wired-connected Profinet network. As the next step, the wired connection was changed to a wireless connection in two standards: 802.11b (2.4 GHz) and 802.11n (5 GHz). The last step was to add an OPC UA server to the network on the AGV side and the OPC UA clients on the Machine side. For each step, the Profinet operation was examined using the Profinet Inspector.

In the considered scenario, the wired Profinet network could operate with update cycles as low as 2 ms with a jitter of about 10%. In contrast to the wired communication, using the wireless 2.4 GHz (802.11b) connection, the Profinet communication was only possible after the update time was incremented to 64 ms. The jitter was more than 70%. According to the PI recommendations, the jitter in Profinet networks should be kept below 20–25%. By increasing the update time to 128 ms, the jitter was reduced to 14%. When the 5 GHz (802.11n) wireless communication was used, the 16 ms update time had a jitter of about 80%. Therefore, for further research, the update time was set to 64 ms with a jitter of less than 20%. Table 2 presents the jitter that was measured for the different scenarios. The cells marked with “---” indicate cases in which real-time communication was not possible for a period that was longer than dozens of seconds.

By default, the real-time data exchange in a Profinet network is considered to be “broken” when three consecutive datagrams are lost or are not sent in time, i.e., are not sent during the expected period of time for the transfer of three datagrams according to the defined update time. When the update time is set to 4 ms, one datagram is sent from the IO-Controller to the IO-Device every 4 ms and one datagram is sent back, If the data exchange is not successfully completed within three IO update cycles (here: 3·4 ms), the communication is considered to be faulty (“broken”). In other words, when the wireless interface introduces a delay that is greater than 12 ms then the Profinet operation is broken.

### 6.4. OPC Communication

The OPC UA server that is implemented on an AGV provides both the individual process variables, which may describe, for example, the operation of the drives, the navigation system, the safety system, the travel routes, etc., and the metadata that describes the type of data. In addition, the aggregated data-blocks that are available on the OPC UA server contain information that is exchanged between the server and a number of systems such as the PLC controller that is mounted on the AGV or the navigation PC that is working in the Simultaneous Localization and Mapping SLAM [46] mode or the data processing blocks that are used by BI systems for edge computing. The latter type of data was used in this experiment.

During the research, the OPC UA server was installed on an industrial PC, which was placed on an AGV. It was created using a Unified Automation C# SDK. It collected data from the AGV, which was organized into 30 blocks of 900 bytes each. The data was accessed by external BI systems (OPC UA clients) to perform the AI and data mining tasks.

The generic OPC UA client software UAExpert (See: https://www.unified-automation.com/products/development-tools/uaexpert.html (accessed on 7 December 2022)) was used during the experiment. In real operating manufacturing systems, the OPC UA clients are typically embedded in the BI software, however in order to simplify the experiment, they were here replaced by several clients that were running as UAExpert applications. This enabled feasible tracking of the transmission parameters since the communication scenario was configured via the explicit configuration of UAExpert. One submission was created for each instance of an OPC UA client. The key parameters for each submission (see Section 3) were as follows:

- requestedPublishingInterval—100 ms,
- requestedLifetimeCount—200 ms and
- requestedMaxKeepAliveCount—1000 ms.

Each data block contained an additional time stamp, which was given by the edge processing systems, and therefore, the value of the parameter requestedLifetimeCount had no effect on the result of the experiment. However, the sampling interval, which was 10 ms for each data block required changes to be made in the data buffering with a queue length of ten samples (see Figure 3).

The parameters stated above were set by the relevant OPC UA session and monitoring item services. In this way, each subscription update was sent every 100 ms and contained 900·30·10 = 270,000 bytes of payload. The additional overhead resulted from the opc.tcp protocol and the need to exchange the segment data for each notification. Each piece of subscription data had to be broken down into 176 Ethernet data frames, which were transmitted every 100 ms with additional frame transitions that were caused by the need to send the Publish Request signals (see Figure 2).

During the experiment, the number of clients connected to the OPC UA server was adjusted from zero to twelve. The connected clients provided an additional communication load. Each of them referred to a separate analytical module associated with the necessary data from the OPC UA server. The clients were located on six desktop PCs that were connected to a wired Ethernet network.

## 7. Results

In the initial phase of the experiments, the minimum update time that could be set in the IO-Devices to enable undisturbed communication depending on the network architecture that was used was determined. As was stated in the previous section and summarized in Table 2, it was found that when using the IEEE 802.11b (2.4 GHz) communication standard, the minimal update time was 128 ms, while for the 802.11n (5 GHz) connection, it was 64 ms. The reason for the different update times is that the data rate of 802.11n is higher than that for 802.11b, and therefore each transmission occupied the medium for a shorter period of time. In addition, the 5 GHz frequency band is generally less populated than the 2.4 GHz frequency band that is frequently used by consumers and industrial systems. When using 5 GHz, the impact of interference is therefore reduced and fewer retransmissions are required. Furthermore, both the shorter transmission range and weaker penetration ability of 5 GHz also contribute to reducing any interference from adjacent areas.

Next, the OPC UA server was started and the jitter in the Profinet communication with a different number of OPC UA clients was measured. The results are summarized in Table 3. There was a slight increase in the jitter when the OPC UA server was started because it exchanged data about the state of the vehicle with the PLC on the AGV side. A more pronounced increase of jitter was also observed when the first OPC UA client was started, which was caused by the fact that the Profinet packets were occasionally forced to wait until an ongoing transmission of an OPC UA packet had been completed, and the OPC UA packets were long, up to 1514 bytes (this applies to our scenario in which the maximum length of Ethernet datagrams was 1522 octets—Ethernet II with VLAN tagging). The great length of the OPC UA packet increased the probability of such a situation and made the consequences more significant—the Profinet packets had to wait a long time to be transmitted.

Another reason for the increased jitter was that because the OPC UA packets were larger, this meant that they occupied the wireless medium for a longer period of time, which increased the probability that some packets were lost due to interference and therefore needed to be retransmitted. Each retransmission added a delay that was dependent on the packet length. In addition, the retransmissions increased the delay due to the binary exponential backoff routine in the carrier sense procedure of IEEE 802.11 because the backoff window size was doubled after each unsuccessful transmission.

However, the largest increase in jitter was observed when the first OPC UA client was added. Adding additional clients did not significantly affect the jitter, see Table 3. The result may not be intuitive since increasing the number of clients increases the number of OPC UA datagrams that are sent in the network. However, the Profinet datagrams had a higher priority than the OPC UA datagrams. Therefore, the Profinet packets were sent before the OPC UA datagrams that were waiting in the queue of a switch. Hence, the number of datagrams in the queues (here primarily the OPC UA datagrams) had a limited impact on the Profinet jitter.

To conclude, the jitter did not substantially increase with the number of clients because the Profinet packets had a higher priority than the OPC UA packets. However, there was still an increase in the jitter in the results because the number of OPC UA packets increased. The reason was that the Profinet packets had to wait for the ongoing transmission of the OPC UA packets to be completed. However, it could be expected that when the tests are performed for a longer period, e.g., a couple of hours or days (The duration of the experiment for each scenario was 20 min), there will only be a limited increase in jitter when the number of OPC UA clients increases above one.

Table 4 presents some of the statistics for the network traffic during the measurements. The traffic was measured using Profinet Inspector. The number of IPv4 datagrams primarily refers to the OPC UA communication, Real-time data transmission in a Profinet network does not use the IP protocol. The number of Profinet data bytes that were received did not change since it depended only on the update time, which was constant, and not on the number of OPC UA clients.

The amount of space and energy on an AGV is limited. Therefore, it is important to use compact and energy-efficient solutions. Additional measurements with the SCALANCE switches, which had been excluded from the testbed, were performed as these switches are quite energy consuming. These switches can be replaced by more energy-efficient devices or even eliminated (see Figure 4). In these measurements, the Profinet devices were connected directly to the EKI switches.

Without the SCALANCE switches, no change was noticed in the jitter parameter as long as there was no traffic other than Profinet. However, there was a change in the jitter value when the OPC UA clients were present in the network. The jitter increased substantially each time a new client was added. This was caused by the fact that the Profinet packets were no longer prioritized in the packet queue in the SCALANCE switches (as they were no longer present in the networks) and were therefore forced to wait until all of the OPC UA packets that had arrived before them had been successfully transmitted. In other words, the EKI switches to which the Profinet devices were connected did not provide an appropriate quality of service. The results are presented in Table 5.

By excluding the SCALANCE switches from the network, the prioritization of the Profinet traffic was no longer performed. This increased the jitter significantly. The above principle of operation is not only applied to the SCALANCE switches, but also to the other switches using the implemented Profinet protocol stack as well as to the other switches that prioritized the datagrams according to IEEE 802.1Q.

## 8. Conclusions

The presented research work was initiated by the need to connect CoBotAGVs with MES and TMS systems in practical applications. In the system that was implemented, two real-time protocols were required to coexist in one wireless network—Profinet for the exchange of the process data and OPC UA for communicating with the higher-level systems. Experimental tests were performed to investigate the challenges associated with combining two communication protocols over a common medium. This challenge has been investigated in the context of wired networks, but to the best of our knowledge, the issue has not been investigated for wireless networks. This industrial research was inspired by Autonomous Guided Vehicles (AGV), which are used in manufacturing not only for transporting materials, semi-finished products, and/or products but also for providing production support via their cooperation with industrial robots. The first context of the use of AGV requires the different communication services that are used by Manufacturing Execution Systems (MES) or Business Intelligence (BI) systems. The second one (cooperation with production stands) requires communication between the control systems that are used at production stations and the systems that are onboard AGV including the vehicle control system and the cooperative robot that is installed on the AGV.

Since AGVs require the use of wireless communication, the shared communication medium is Wi-Fi as it is closest to Ethernet, which is widely used in industrial solutions. The research focused on sharing wireless communication between the OPC UA protocol for communicating with MES systems and the Profinet I/O protocol for sending real-time data. The research began with wired communication, which, while it is not possible when using AGV, was used as a benchmark in order to compare communication in wired media and communication in wireless networks. The research was conducted for a fixed configuration of machine-to-machine (M2M) communication that was established via Profinet network (the configuration for a given production station resulted directly from the architecture of the control system including the number of I/O modules) and machine-to-system (M2S) communication with a variable number of OPC UA clients. Each client corresponded with the MES or TMS services by requesting data from the AGV. The number of services that were provided at a given moment varied and depended on the tasks that were being performed at the MES level. The quality of the Profinet communication network was analyzed by measuring the jitter as a parameter that was connected with the number of OPC UA subscriptions.

In the next step, the same assumptions were held for an 802.11b (2.4 GHz) wireless network. In the third step, the 2.4 GHz wireless network was replaced with an 802.11n (5 GHz) network. In all of the above cases, on the side of the AGV and the production station, the wired network segments were based on a dedicated SCALANCE switch (with the Profinet protocol stack implemented in it. In order to ensure operation in accordance with the mechanisms that were specified in Section 2). In the last experiment, the SCALANCE switches were removed from the system and the Ethernet connections of Profinet devices were provided directly to the wireless EKI switches.

The experimental results that are described in Section 5 led to the following research conclusions: (i) replacing the wired network with the wireless segment required that the appropriate frequencies for the information exchange between the PLC and I/O devices had to be selected by the appropriate parameterization of the Profinet network. If the exchange periods were too short, there was a complete breakdown of communication in the Profinet network as is shown in Table 2. Since the performed research did not include any methods to automatically determine these parameters, they suggest the direction of further work in this area; (ii) a change in the number of OPC UA clients did not significantly affect the parameters of this network. A comparison of the 2.4 GHz and 5 GHz wireless communication also did not indicate any significant differences in the scope of the research that was analyzed (apart from the change in the update time); (iii) replacing the switches that were dedicated to the Profinet network with general-purpose Ethernet switches resulted in the degradation of the temporal characteristics of the real-time communication channels, which was caused by the increased OPC UA communication traffic; and (iv) the OPC UA communication is resistant to load changes and can be used within the scope of soft real-time communication with loads that are changeable. During the research, information was exchanged for all of the subscriptions (from 1 to 12) during a period of 100 ms and no breakdown in communication was observed. The result was obtained using a requestedLifetimeCount OPC UA parameter of 1 s. The OPC UA protocol did permit continuous operation in the subscription mode with a variable load during the entire experiment.

The main contribution of the paper is that the presented experimental test shows the applicability of the independent real-time communication protocols in one wireless interface. Although the OPC UA data exchange influenced the Profinet data exchange by increasing its jitter, both communication protocols can coexist in one network. Increasing the OPC UA-generated netload had a limited impact on the result. It was also shown that the number of OPC UA clients did not decrease the temporal parameters of the Profinet communication significantly. In addition, our experiments showed that using switching devices with the Profinet protocol stack implemented is recommended as it improves the quality of the Profinet characteristics.

The authors believe that the presented research will be helpful for designing industrial communication systems that have to share the communication medium between control systems and higher-level systems (MES, TMS, others) as it is presented in the use case with the AGV that are used in flexible manufacturing and that are simultaneously connected by OPC UA and Profinet that are based on wireless networks. However, further research is required to automatically calculate wireless communication parameters that can change during the operations that are performed by AGV.

## Figures and Tables

**Table 1:** Attenuation in different material types.

Material	Thickness [cm]	Attenuation [dB]
Brick	30	9
	10	7
Concrete	30	11
Wood	4	2.5
Glass	2	4.5

**Table 2:** Starting configurations of the experimental testbeds.

Testbed Configuration		Update Time *			
		2 ms	4 ms	64 ms	128 ms
Wired connection	jitter	10.7%	4.78%	0.1%	~0.1%
	jitter [ms]	0.2	0.19	0.06	~0.06
Wireless 2.4 GHz (b)	jitter	---	---	---	14.1%
	jitter [ms]	---	---	---	18.0
Wireless 5 GHz (n)	jitter	---	---	18.8%	7.6%
	jitter [ms]	---	---	12.0	9.72

* “---“ indicates a lack of communication.

**Table 3:** Results for the wireless connection with an update time of 128 ms.

Measurement Setup	2.4 GHz		5 GHz	
	Jitter %	Jitter [ms]	Jitter %	Jitter [ms]
No OPC UA server	2.4%	3.0	14.1%	18.0
OPC UA server active *	3.2%	4.1	20.4%	26.1
1 client on 1 PC	3.7%	4.7	39.2%	50.2
2 clients on 2 PCs	4.4%	5.6	32.5%	41.6
4 clients on 4 PCs	5.5%	7.0	35.7%	45.7
6 clients on 6 PCs	6.8%	8.7	37.0%	47.4
8 clients on 6 PCs	*7.3%*	*9.3*	*38.9%*	49.8
10 clients on 6 PCs	*8.5%*	10.9	*40.2%*	51.5
12 clients on 6 PCs	*10.8%*	13,8	*41.8%*	53.5

* only background communication with the AGV.

**Table 4:** The number of IPv4 datagrams and the size of the data that was sent and received depending on the number of OPC UA clients.

Measurement Setup	No. of IPv4 Datagrams	MBytes Sent		MBytes Received	
		Total	Profinet	Total	Profinet
UPC server, no clients	80	0.41	0.37	0.37	0.37
1 client, 1 PC	5064	2.26	0.37	2.20	0.37
2 clients, 2 PCs	7376	3.90	0.37	3.85	0.37
4 clients, 4 PCs	11,928	7.21	0.37	7.17	0.37
6 clients, 6 PCs	16,351	10.52	0.37	10.48	0.37
8 clients, 6 PCs	26,582	13.43	0.37	13.39	0.37
10 clients, 6 PCs	35,457	17.71	0.37	17.69	0.37

**Table 5:** Results for the 2.4 GHz wireless connection without the SCALANCE switches with a 128 ms update time.

Measurement Setup	Total	Profinet
No OPC UA server	12.6%	16.1
UPC UA server active, no clients	24.5%	31.4
1 client on 1 PC	23.4%	29.9
2 clients on 2 PCs	37.6%	48.1
3 clients on 2 PCs	65.9%	84.4
4 clients on 2 PCs	110.5%	141.4
